# Explainable AI reveals temporal risk pathways in fall prediction: Extracting clinical insights from multi-horizon machine learning models

**DOI:** 10.1007/s11357-026-02117-x

**Published:** 2026-02-04

**Authors:** Masoud Khani, David R. Friedland, Michael Widlansky, Michael S. Harris, Jazzmyne Adams, Lei Fan, Hyunkyoung Oh, Qiang Lu, Jake Luo

**Affiliations:** 1 Department of Health Informatics & Computer Science, University of Wisconsin-Milwaukee, Milwaukee, WI USA; 2 Keck School of Medicine, University of Southern California, Los Angeles, CA USA; 3 Department of Otolaryngology & Communication Sciences, Medical College of Wisconsin, Milwaukee, WI USA; 4 Department of Biomedical Engineering, Marquette University, Milwaukee, WI USA; 5 School of Nursing, University of Wisconsin-Milwaukee, Milwaukee, WI USA; 6 Department of Computer Science & Technology, University of Petroleum, Beijing, China

**Keywords:** Fall risk prediction, Explainable AI (XAI), Electronic health records (EHRs), Temporal risk modeling, Machine learning in healthcare, Geriatric syndromes

## Abstract

Falls are a leading cause of injury in older adults, making risk prediction a clinical priority. While many machine learning (ML) models exist, they typically provide a static assessment, predicting risk for a single, fixed timeframe. This approach overlooks how the clinical drivers of risk evolve over time, failing to distinguish between acute factors that signal an imminent fall and chronic conditions that confer long-term vulnerability. This study uses an explainable ML framework across multiple time horizons to unlock deeper clinical insights into the temporal nature of fall risk. We conducted a retrospective, matched case-control study using electronic health record (EHR) data from 99,078 patients who fell and 99,078 matched controls. Seven ML models were trained to predict fall risk across seven distinct prediction windows (3, 6, 12, 24, 36, 48, and 60 months). The best-performing model for each horizon, consistently XGBoost, was interpreted using SHAP (SHapley Additive exPlanations) to identify how the importance of clinical and demographic predictors changed over time. A clear performance trade-off emerged across time horizons. Short-term models (3–12 months) delivered balanced discrimination (best model XGBoost, AUC ≈ 0.75), while long-term models became progressively better at identifying eventual fallers (recall ≈ 80% at 60 months) at the cost of lower specificity (≈ 46%). SHAP analysis revealed distinct temporal patterns: short-term risk was driven by acute conditions like syncope, respiratory symptoms, and urinary tract infections, while long-term risk was predicted by chronic, cumulative factors such as spondylopathies, nutritional deficiencies, and benign neoplasms. Three primary risk trajectories (increasing, steady, and decreasing) were identified, each corresponding to distinct underlying clinical profiles. Fall risk is a dynamic process, not a static state. By analyzing risk across multiple timeframes, we can distinguish between acute triggers requiring immediate intervention and chronic vulnerabilities demanding long-term management. This multi-horizon framework provides a data-driven foundation for a new paradigm in fall prevention: moving beyond generic “high-risk” labels to personalized, temporally aware strategies that align the type and timing of interventions with the specific nature of a patient’s evolving risk.

**Supplementary information:**

The online version contains supplementary material available at https://doi.org/10.1007/s11357-026-02117-x.

## Introduction

Falls represent a major healthcare challenge, being the leading cause of injury-related complications in older adults [1]. Epidemiological data show that about 30% of individuals aged 65 years and older experience falls annually, rising to 40% for those over 80 years [2, 3]. These events often lead to adverse clinical outcomes, from minor injuries to severe trauma, prolonged disability, loss of independence, and increased mortality [2, 4]. The economic burden is substantial, with annual US healthcare costs exceeding $50 billion [5].

Effective fall prevention requires accurate identification of at-risk individuals for targeted interventions [6]. Traditional approaches rely on clinical assessments, tools like the Morse Fall Scale [7] and STRATIFY [8], and multifactorial risk scoring [9]. However, these methods have key limitations. They often use binary (high/low) classifications, lacking nuance [10], and rely on single time-point data while failing to account for variations in modifiable risk factors, resulting in poor predictive performance and an inability to track the evolving nature of fall risk over time [11]. In addition, many hospital screening tools are limited because they include questions about whether a patient has already fallen, an indicator that is post-event and therefore less useful for prevention [7, 8].

Recent advances in machine learning (ML) have significantly advanced fall risk prediction in older adults by addressing the limitations of traditional methods through the integration of diverse data sources and advanced algorithms. ML models effectively process large, multivariate datasets, including electronic health records (EHRs) containing features like fall history, medications, psychiatric conditions, mobility scores, and pain levels, as well as sensor-derived data from wearables such as accelerometers, gyroscopes, and inertial measurement units (IMUs) for gait metrics like stride variability, acceleration, angular velocity, and Center of Pressure (COP) [12–18]. Common supervised ML techniques include logistic regression, decision trees, random forests, support vector machines (SVMs), k-nearest neighbors (KNN), gradient boosting (e.g., XGBoost), and AdaBoost for classifying fallers using EHR or clinical assessment data in hospital, long-term care, or community settings, often achieving area under the curve (AUC) of 0.70–0.87 [19, 20]. For sensor-based approaches, deep learning methods like convolutional neural networks (CNNs) and bidirectional long short-term memory (BLSTM) networks analyze temporal gait features from tests such as Timed Up and Go (TUG) or daily activities, yielding high sensitivity (up to 86%) in identifying fall-prone individuals in prospective cohorts [14, 16, 21, 22]. While these wearable sensors are valuable for detecting real-time mechanical instability, they primarily capture proximal gait disturbances and depend on consistent device use. In contrast, EHR-based prediction leverages the rich, longitudinal record of diagnoses, medications, and comorbidities to reveal underlying systemic vulnerability before overt physical decline, thereby supporting more preventive, population-level risk stratification. Additionally, explainable AI (XAI) techniques, such as SHAP (SHapley Additive exPlanations) [23] values and feature importance analysis, enhance transparency by highlighting key predictors and linking predictive power with clinical insights [15, 24].

Although different studies have employed various machine learning approaches to predict falls in older adults, their focus is typically on short term or near the event outcomes. This creates a critical gap, highlighting the need for advanced AI-driven approaches that consider how fall risk evolves over long-term dynamic timelines [11, 25]. Most studies assess risk over fixed, short windows, assuming constant risk factors and biasing toward signals near the event or predicting the recurrent fall by relying on previous fall history as a key predictor, which inherently focuses on forecasting recurrent falls rather than preventing the initial one [13, 15–17, 26]. This retrospective focus limits true prospective forecasting and fails to address key questions: (1) Do predictors of imminent falls (e.g., within months) differ from those years ahead? and (2) How do acute health conditions versus chronic changes influence risk? Without examining risk evolution over extended periods, interventions remain generalized rather than personalized.

Based on the two research questions, we developed a novel EHR-based ML model designed for integration into clinical information systems to comprehensively estimate and monitor fall risk for preventive care. By employing ML and XAI to analyze feature relationships with the risk of falling across different time frames, this research facilitates a transition from static, binary risk labels to dynamic, multi-horizon risk profiles. By analyzing these data, we can extract more insights from the ML models and better comprehend the temporal dynamics of disease-related outcomes. Some features may exhibit short-term effects, while others may have long-term implications. This distinction is crucial for clinical utility. By understanding whether a patient’s risk is driven by short-term acute factors or long-term chronic deterioration, physicians can move beyond generic warnings to targeted patient education and individualized preventative care plans, informed directly by the EHR-derived risk trajectories.

The structure of this paper is as follows: the “Methodology” section details our data source, feature selection, ML methodology, and evaluation metrics. The “Results” section compares model performance across time horizons and examines evolving feature importance. The “Discussion” section discusses clinical implications, strengths, limitations, and future directions. The “Conclusion” section summarizes key findings and their broader impact on fall prevention and healthcare.

## Methodology

**Fig. 1:**
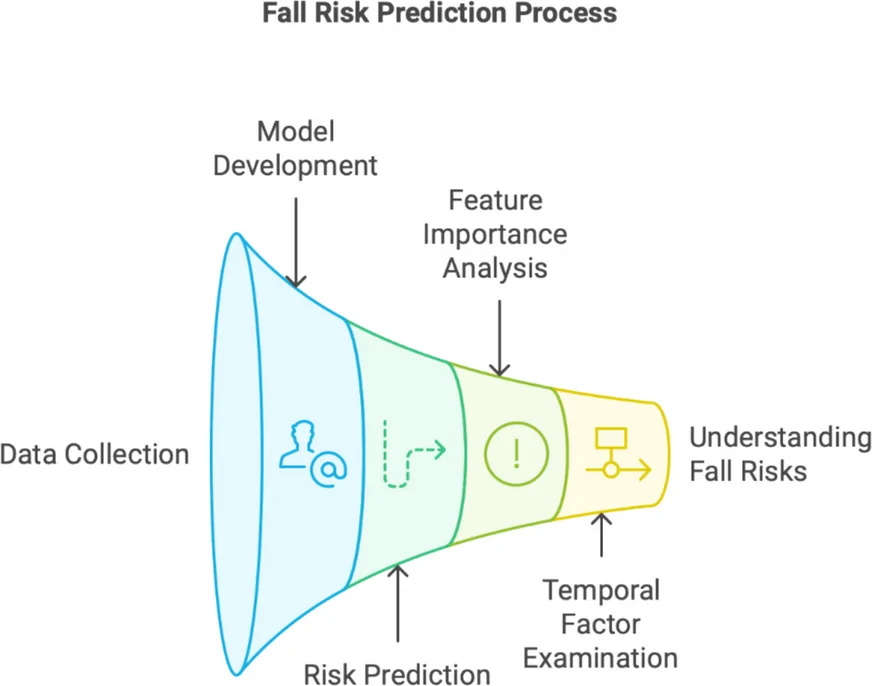
Fall risk analysis pipeline. The process begins with comprehensive data collection, progresses through model development and feature importance analysis, and incorporates temporal factor examination to enhance predictive accuracy. The workflow culminates in risk prediction capabilities and a comprehensive understanding of fall risks, showing the systematic progression from initial data gathering to actionable risk assessment outcomes

The methodology was designed as a systematic pipeline encompassing data collection, preprocessing, feature engineering, model development, and validation phases, as illustrated in Fig. 1. The analytical framework integrated multiple data sources and employed advanced statistical and ML techniques to identify key predictors of fall risk while accounting for temporal factors that influence fall occurrence.

### Data source and study population

This study employed a retrospective, matched case-control design using de-identified EHR data from the Froedtert Hospital system in Milwaukee, WI, USA. The dataset included all patients who had a clinical encounter between October 1, 2015, and December 31, 2024. The study was conducted in accordance with the Declaration of Helsinki and received approval from the Froedtert & the Medical College of Wisconsin Institutional Review Board (IRB), which granted a waiver of informed consent due to the retrospective and de-identified nature of the data (IRB# PRO00045781).

Cases were identified as patients who experienced at least one documented fall during the study period. A fall event was identified using International Classification of Diseases, Tenth Revision (ICD-10) codes related to falls (e.g., W00–W19). For each case, the date of their first documented fall was defined as the index date. Inclusion criteria required patients to be at least 18 years of age at the time of the index event. In this adult cohort (median age 57 years), 45% of patients met the standard definition of “older adult” (≥ 65 years), indicating substantial representation of the geriatric population. We deliberately adopted this broader age range to enhance generalizability across the adult lifespan and to capture latent risk factors and early disease trajectories that may precede clinically apparent geriatric syndromes. This life-course perspective is particularly relevant for geriatric care, as it enables the development of EHR-based tools that can flag high-risk individuals earlier and support targeted fall prevention strategies before disability or recurrent falls become established.

The control group consisted of patients with no documented history of falls within the EHR system. For each case, a unique control was selected from this population using a 1:1 matching procedure. Matching was performed based on age and gender. Priority was given to matching age as closely as possible. A match on gender (male/female) was subsequently performed. This matching strategy is crucial for controlling the powerful confounding effects of age and sex. However, complete matching across additional demographic variables such as gender, race, and ethnicity was not feasible due to limitations in cohort size, which prevented exact alignment across all categories. In addition to matching, demographic variables (age, sex, race, and ethnicity) were retained as predictors in the ML models so that fall risk could be estimated conditional on both clinical and sociodemographic context and so that residual differences across groups could be examined. We interpret these variables as potential markers of structural and access-related inequities rather than inherent biological susceptibility, and we view subgroup-specific performance assessment as an important step toward evaluating the fairness of any future clinical deployment.

By ensuring the case and control groups are demographically similar, we can be more confident that the differences identified by our models are attributable to clinical factors rather than proven baseline demographics. Table 1 presents the demographic characteristics of the study population, comparing the fall group to the control group. The analysis included a total of 198,156 patients, with 99,078 patients in each cohort.

**Table 1:** Demographic analysis

	Fall		Control		Odds ratio
Number of patients	99,078		99,078		
Median age (controlled)	57.28 [27, 28]		57.28 [27, 28]		
Sex	*N*	%	*N*	%	
Male	41,868	42.26%	47,037	47.47%	0.81
Female	57,206	57.74%	51,927	52.41%	1.241
Race					
White or Caucasian	74,107	74.80%	64,482	65.08%	1.592
Black or African American	17,855	18.02%	11,757	11.87%	1.633
Asian	985	0.99%	1801	1.82%	0.542
Unknown	411	0.41%	4289	4.33%	0.092
American Indian or Alaska Native	367	0.37%	331	0.33%	1.109
Patient refused	116	0.12%	272	0.27%	0.426
Multiracial	303	0.31%	373	0.38%	0.812
Native Hawaiian or Other Pacific Islander	39	0.04%	72	0.07%	0.541
Ethnicity					
Not Hispanic or Latino	93,322	94.19%	77,341	78.06%	4.557
Hispanic or Latino	3955	3.99%	4507	4.55%	0.872

A notable difference was observed in the distribution of sex. Females were more prevalent in the fall group (57.74%) compared to the control group (52.41%), with an odds ratio (OR) of 1.241, suggesting a higher likelihood of being in the fall group. Regarding race, individuals identifying as White or Caucasian (74.80% vs. 65.08%; OR = 1.592) and Black or African American (18.02% vs. 11.87%; OR = 1.633) constituted a larger proportion of the fall group compared to the controls. The most significant difference was seen in ethnicity. Patients who were “Not Hispanic or Latino” were substantially more represented in the fall group (94.19%) than in the control group (78.06%), which is reflected in a high odds ratio of 4.557. Conversely, those identifying as “Hispanic or Latino” were less frequent in the fall group (3.99% vs. 4.55%; OR = 0.872).

### Data collection and preprocessing

The predictor variables in this study were derived from patients’ diagnostic histories as documented in ICD-10 codes. To examine how fall risk predictors evolve across different time spans, we built seven temporally distinct datasets, rather than relying on a single static feature set. Each dataset corresponded to one of the designated prediction windows: 3, 6, 12, 24, 36, 48, and 60 months. These intervals were chosen to mirror how risk changes are monitored in practice: the 3-, 6-, and 12-month windows provide finer resolution over the short term, when recent diagnoses, exacerbations, and medication changes are most likely to alter fall risk, whereas beyond 12 months we used yearly windows because comorbidities and functional decline typically evolve more gradually over years rather than months. The look-back period was capped at 5 years (60 months) to ensure that predictors remained clinically relevant and well-documented.

**Fig. 2:**
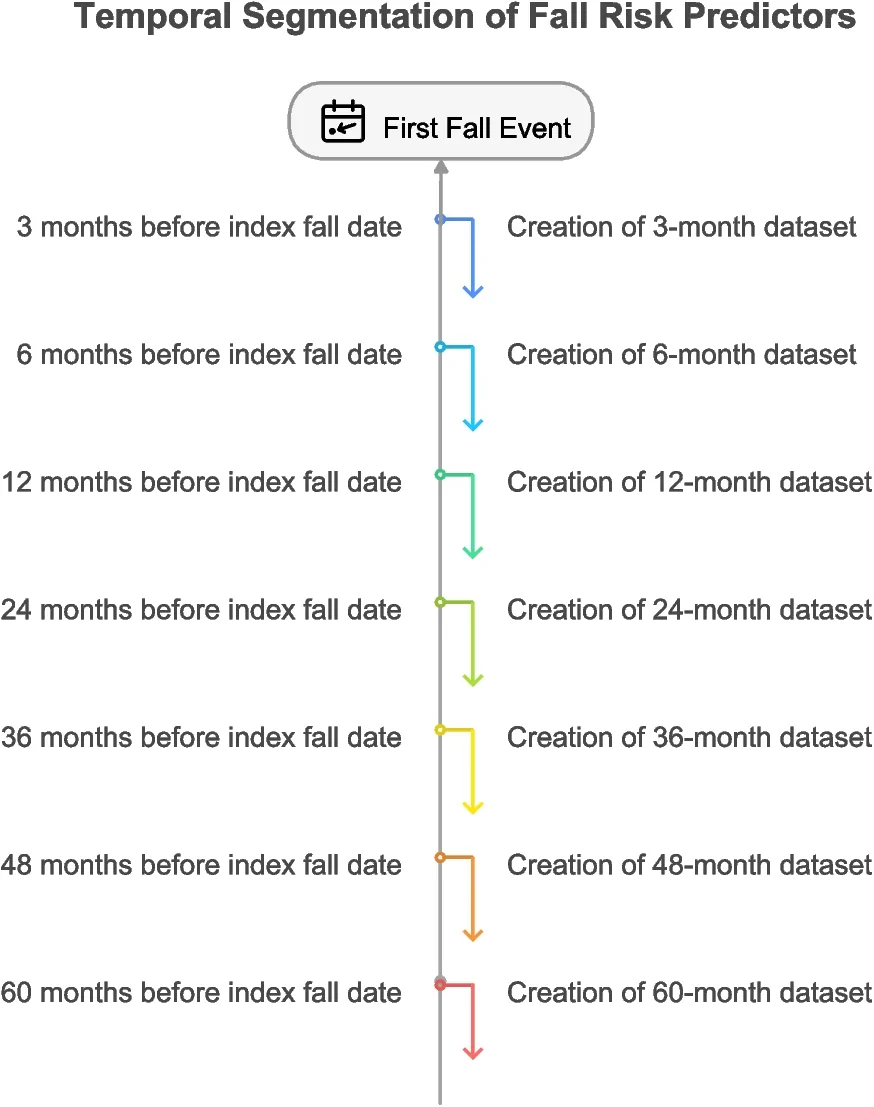
Temporal segmentation of fall risk predictors. The process segments historical patient data into multiple time windows ranging from 3 to 60 months prior to the first fall event. Each temporal segment generates a corresponding dataset, enabling the analysis of fall risk predictors across different time horizons and supporting the development of predictive models with varying prediction windows

Dataset construction was deliberately constrained by time. For example, in the 3-month horizon, a case patient’s dataset included all diagnoses recorded during the 3 months preceding the index fall. For the matched control, we identified their most recent diagnosis within the study period and then collected codes from the 3 months prior to that point. This anchoring ensured that controls were evaluated relative to their own clinical trajectory rather than the case’s timeline. The same procedure was applied consistently across all seven horizons. Figure 2 illustrates this temporal segmentation strategy, showing how datasets were constructed at multiple predefined intervals before the index fall event.

Such temporal segmentation was central to the study design because it minimized the risk of information leakage. Each prediction model was trained only on data that would have been available within its respective timeframe. In practice, this means the 3-month model, for instance, had no access to diagnoses recorded 4 months before the index event. This approach not only reduces bias but also allows for a clearer picture of how the predictive value of clinical features shifts from short- to long-term windows.

### Preprocessing

Following the construction of the seven temporal datasets, a multi-step preprocessing pipeline was applied to prepare the data for machine learning analysis. Each dataset was processed independently to preserve its temporal integrity.

1. *Diagnosis code aggregation using CCSR*: The raw predictor variables consisted of thousands of unique ICD-10 codes. To create clinically meaningful and computationally manageable features, we mapped these ICD-10 codes to the Clinical Classifications Software Refined (CCSR) [29] for ICD-10. The CCSR system aggregates individual diagnosis codes into a smaller number of clinically coherent categories (e.g., individual codes for various types of hypertension are grouped into a single “Hypertension” category). This transformation offers two key advantages for our analysis: first, it significantly reduces the dimensionality of the feature space, which helps mitigate the risk of overfitting; second, it consolidates related diagnoses into single, powerful predictors that better represent a patient’s overall clinical condition, enhancing the interpretability of the model’s findings.
2. *Imputation of missing values*: Missing values were present in a subset of the predictor variables. To handle this, we employed an imputation strategy tailored to each specific time horizon. For each of the seven datasets, a separate random forest imputation model was trained. This approach was chosen over a single global imputation model because the relationships and predictive power of variables can differ significantly across short-term versus long-term windows. By training a unique imputer for each dataset, we ensure that the imputed values are based on the correlational structures most relevant to that specific timeframe, leading to a more accurate and context-aware data representation.
3. *Feature scaling*: After imputation, all numerical features in the datasets were scaled to a uniform range between 0 and 1 using the Min-Max Scaler. This scaling is essential for many ML algorithms, including logistic regression and other models sensitive to the magnitude of input features. Normalizing the feature scales ensures that no single variable can disproportionately influence the model’s learning process simply due to its larger numerical range, thereby allowing the models to converge more efficiently and weigh the importance of each predictor based on its actual contribution to fall risk.

### Machine learning algorithms development

To comprehensively evaluate fall risk prediction across different time horizons, we developed and compared a diverse suite of seven ML algorithms, with each model being trained and evaluated independently on each of the seven temporal datasets. Our selection began with logistic regression, a well-established linear model that serves as an interpretable baseline. We then included a single decision tree for its transparent, rule-based logic. To leverage the power of ensemble methods, we implemented two bagging-based algorithms: the widely used random forest, which improves accuracy and controls overfitting, and its variant, extra trees (extremely randomized trees), which introduces additional randomness to potentially reduce variance. Finally, our approach incorporated three powerful boosting algorithms that build models sequentially: gradient boosting, which trains new models to correct the errors of previous ones; AdaBoost (adaptive boosting), which adaptively focuses on difficult-to-classify instances; and XGBoost (extreme gradient boosting), a high-performance framework renowned for its speed, scalability, and state-of-the-art predictive accuracy.

### Model training, validation, and evaluation

For each of the seven prediction windows, the corresponding dataset was partitioned into a training set (80%) and a held-out testing set (20%) using a patient-wise stratified split. This method ensures that all records for a given patient belong exclusively to either the training or testing set, which prevents data leakage. Furthermore, the stratification maintains the same 1:1 ratio of fall cases to non-fall controls in both the training and testing partitions, ensuring a balanced evaluation.

To ensure robust model development, we performed hyperparameter tuning on the training data using a fivefold cross-validation scheme coupled with a randomized search strategy. The optimal hyperparameter combination for each algorithm was selected based on the configuration that yielded the highest area under the receiver operating characteristic curve (AUC) during cross-validation. Once finalized, the models were trained on the entire training set, and their ultimate performance was assessed on the unseen test set. This final evaluation was conducted using a comprehensive suite of standard classification metrics, including AUC as the primary measure of discriminative ability, alongside accuracy, precision, recall (sensitivity), F1-score, and specificity. This entire process was repeated for all seven models across all seven-time horizons, allowing for a thorough and unbiased comparison of their predictive performance.

### Explainable AI (XAI) and feature importance analysis

To move beyond predictive accuracy and understand the underlying clinical drivers of fall risk, we employed SHAP, an advanced XAI technique. For each of the seven-time horizons, we applied SHAP analysis to the best-performing model, as determined by the highest AUC on the test set. SHAP provides a theoretically sound framework for interpreting model predictions by assigning each feature an importance value for every individual prediction, quantifying both the magnitude and direction of its contribution. We calculated these values for all features across all patients for each of the seven prediction windows. This comprehensive analysis enabled us to investigate the temporal dynamics of fall risk predictors. First, we determined the global feature importance for each time horizon by averaging the absolute SHAP values across all patients. Second, we systematically compared these feature importance rankings across all seven prediction windows to identify how the influence of different clinical factors evolved over time. Based on these temporal trends, we categorized predictors into three distinct groups: **short-term specific** (highly important for 3–12-month windows), **long-term specific** (gaining importance in 24–60-month windows), and **persistent** (maintaining high importance across most or all horizons). This approach provides a granular, interpretable view of the changing nature of fall risk.

## Results

Given the large number of models (7) and prediction windows (7), presenting individual performance tables for all 49 trained models would be impractical. Therefore, to summarize the overall predictive performance, we calculated the mean and standard deviation (mean ± STD) of the key evaluation metrics across all seven models for each time horizon. The aggregated results are presented in Table 2. The results of each timeframe and machine learning are presented in Appendix 1.

**Table 2:** Model performance comparison for fall prediction

Horizon	Accuracy (%)	Precision	Recall/sensitivity	F1-score	AUC	Specificity
3	**65.849 ± 2.232**	**0.655 ± 0.026**	0.668 ± 0.061	0.66 ± 0.029	**0.72 ± 0.05**	**0.649 ± 0.055**
6	65.456 ± 2.101	0.65 ± 0.022	0.666 ± 0.068	0.66 ± 0.032	0.713 ± 0.043	0.643 ± 0.056
12	64.549 ± 2.398	0.637 ± 0.021	0.672 ± 0.071	0.65 ± 0.036	0.704 ± 0.043	0.62 ± 0.049
24	63.646 ± 2.251	0.617 ± 0.018	0.711 ± 0.036	0.66 ± 0.025	0.69 ± 0.04	0.562 ± 0.021
36	63.149 ± 1.948	0.606 ± 0.018	0.749 ± 0.029	0.67 ± 0.017	0.681 ± 0.036	0.515 ± 0.042
48	62.933 ± 1.837	0.6 ± 0.017	0.77 ± 0.028	0.67 ± 0.015	0.675 ± 0.035	0.49 ± 0.042
60	62.861 ± 1.597	0.596 ± 0.018	**0.799 ± 0.049**	**0.682 ± 0.012**	0.669 ± 0.033	0.459 ± 0.07

Values in bold indicate the best (highest-performing) result for each metric

**Fig. 3:**
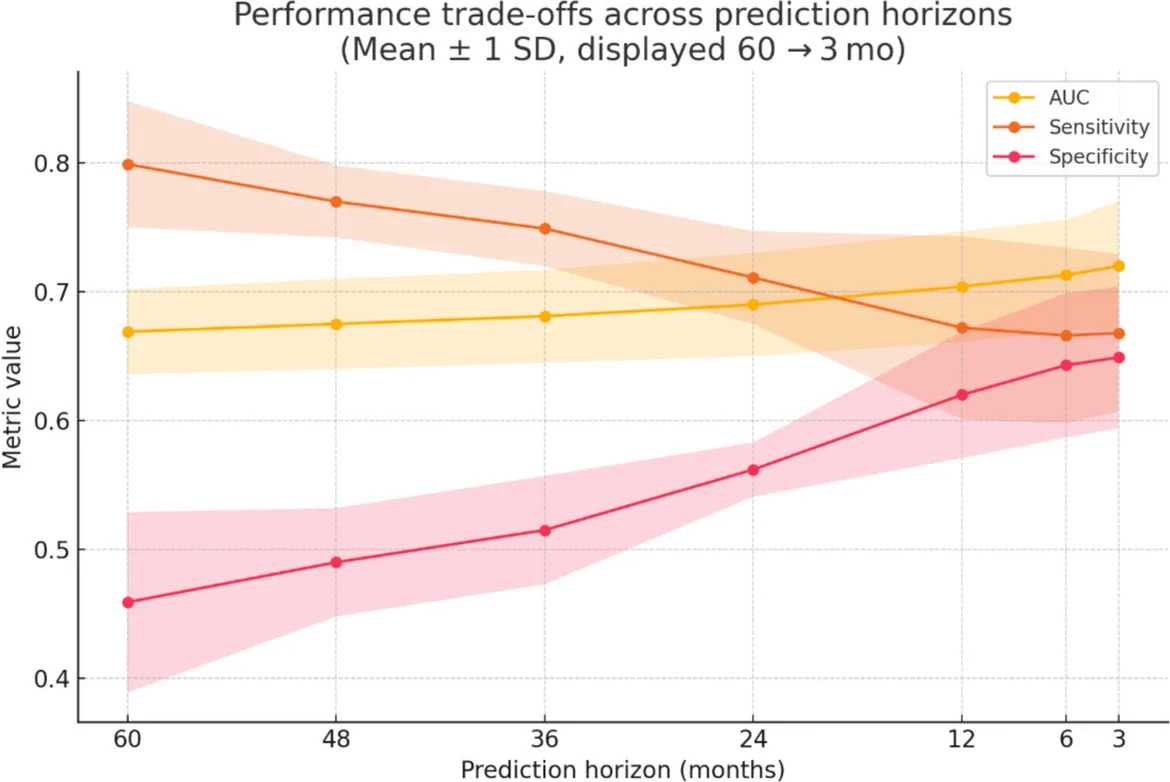
Performance trade-offs across prediction horizons. The graph shows how model performance changes when predicting falls over different time windows (60 to 3 months before the event). To summarize the overall predictive performance, we calculated the mean and standard deviation (mean ± STD) of the key evaluation metrics across all seven models for each time horizon. The central lines represent the mean values, with shaded areas indicating one standard deviation. AUC (orange) declines gradually from 0.72 ± 0.05 at 3 months to 0.669 ± 0.033 at 60 months. Sensitivity/recall (yellow) shows a clear upward trend, rising from 0.668 ± 0.061 at 3 months to 0.799 ± 0.049 at 60 months. Conversely, specificity (pink) demonstrates the opposite pattern, declining from 0.649 ± 0.055 at 3 months to 0.459 ± 0.07 at 60 months. This illustrates the fundamental trade-off where longer prediction horizons achieve higher sensitivity (better at detecting future fallers) at the cost of substantially lower specificity (more false positives)

Our results show a clear trade-off in model performance depending on how far into the future we try to predict falls (Fig. 3). **Short-term prediction models** (predicting falls in the near future) provide the most balanced accuracy. They achieve good overall performance (AUC of 0.70–0.72) with reasonable ability to both identify future fallers and correctly classify non-fallers (sensitivity and specificity both around 63–67%). **Long-term prediction models** (predicting falls further into the future) become increasingly biased toward identifying potential fallers. At 60 months, these models catch about 80% of people who will eventually fall, but the specificity drops to 46%. This happens because when we extend the prediction window, more people in our dataset will have experienced a fall by the end of that period. With more “true positive” cases to learn from, the model becomes better at identifying potential fallers but worse at distinguishing them from non-fallers. Essentially, the model errs on the side of caution, casting a wider net that captures more actual future fallers but also generates more false alarms.

When comparing individual model performances, the XGBoost algorithm consistently emerged as the top-performing model (details in Appendix 1), achieving the highest AUC across all seven-time horizons. At 3 months, XGBoost reached an AUC of 0.7581, outperforming random forest (0.7473) and extra trees (0.7348). At 6 months, it again led with an AUC of 0.7543, followed closely by random forest (0.7421). For the 12-month horizon, XGBoost achieved an AUC of 0.7455, surpassing random forest (0.7344). Performance differences became more pronounced at longer horizons: at 24 months, XGBoost obtained an AUC of 0.7322, higher than random forest (0.7163); at 36 months, it reached 0.7200, ahead of other models, which generally dropped below 0.71. At 48 months, XGBoost maintained an AUC of 0.7125, again leading over random forest (0.7007). Finally, at the 60-month horizon, XGBoost continued to perform best with an AUC of 0.7055, while random forest (0.6923) and gradient boosting (0.6799) trailed behind.

### Explainable AI analysis

**Fig. 4:**
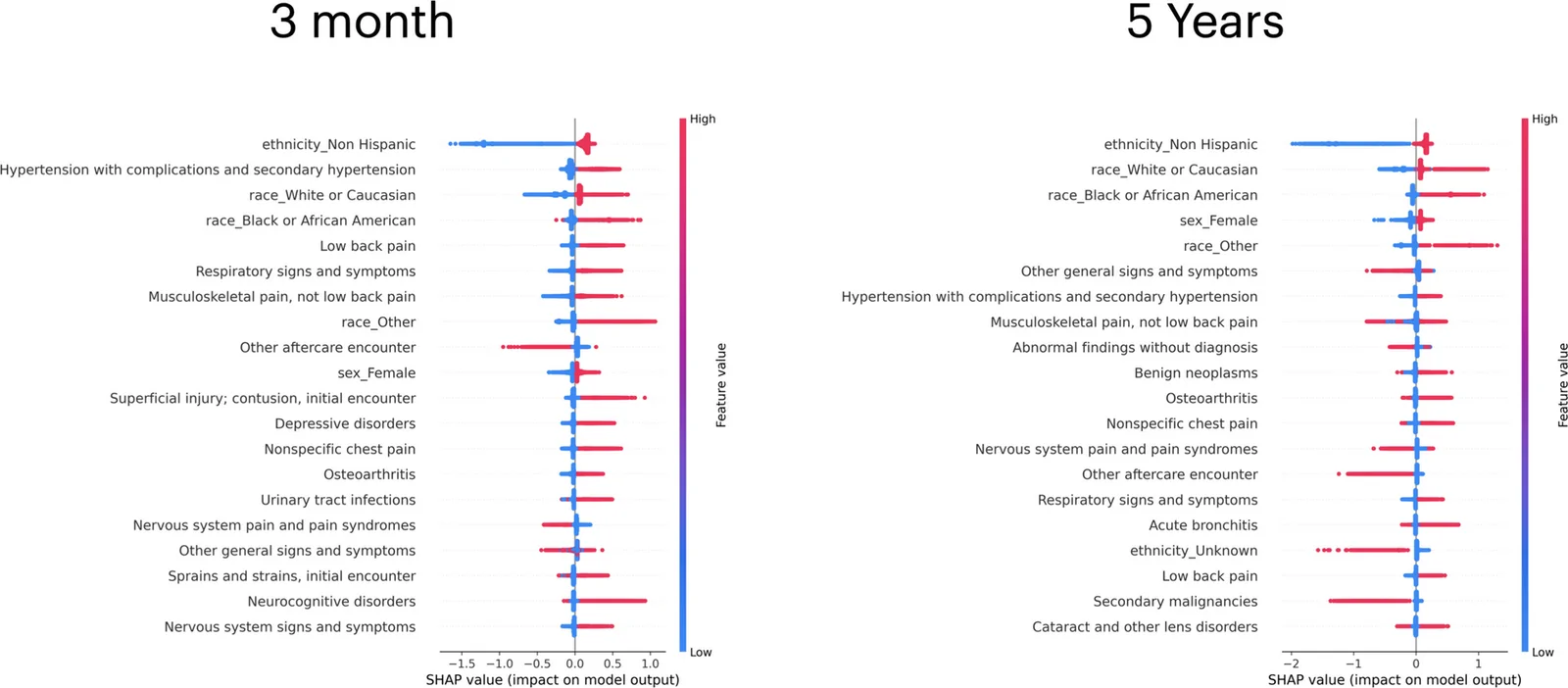
SHAP summary plots comparing feature importance across prediction horizons. The plots show SHAP value distributions for the top predictive features in XGBoost models trained for 3-month (left) and 5-year (right) fall prediction windows. Features are ranked vertically by their overall importance (mean absolute SHAP values), with the *x*-axis representing individual SHAP values where positive values indicate increased fall risk and negative values indicate decreased risk. Each point represents a patient-feature combination, colored from blue (low feature values) to red (high feature values). The plots reveal distinct feature importance patterns between short-term and long-term predictions, demonstrating how the model prioritizes different clinical factors depending on the prediction timeframe. The variation in feature rankings and SHAP value spreads illustrates the temporal evolution of fall risk factors across different prediction horizons

To interpret the XGBoost model predictions, we conducted SHAP analysis to identify key clinical factors driving fall risk at different time horizons. Figure 4 shows SHAP summary plots for short-term (3 months) and long-term (5 years) predictions. Features are ranked by importance (mean absolute SHAP values), with positive values indicating increased fall risk and negative values indicating decreased risk. Point colors represent feature values from low (blue) to high (red).

It is evident that each time horizon utilizes different features for predicting falls, with distinct feature rankings and importance patterns visible between the 3-month and 5-year models. The variation in feature positions and SHAP value distributions demonstrates how the model’s decision-making process adapts to different prediction timeframes, with some features showing greater predictive power for immediate risk while others become more important for long-term predictions. To systematically analyze these temporal changes in feature importance and understand how clinical factors evolve as predictors across all seven-time horizons, we calculated SHAP values across the complete timeline and examined the differences in their predictive contributions.

**Fig. 5:**
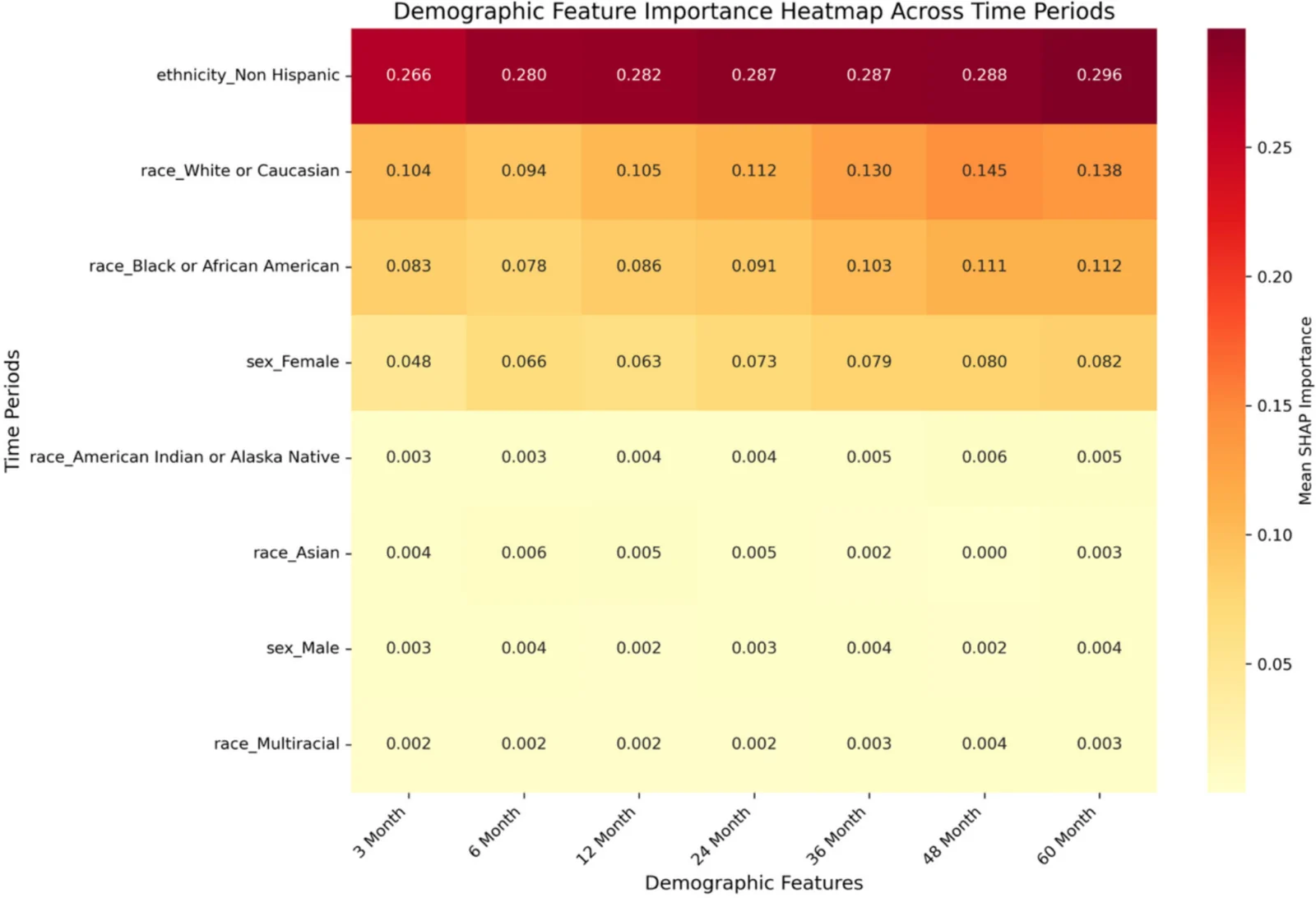
Temporal dynamics of demographic feature importance in fall prediction models. Heatmap displays mean SHAP importance values for demographic predictors across seven prediction windows (3 to 60 months before fall events). Color intensity represents predictive importance magnitude, with darker red indicating higher values. Non-Hispanic ethnicity consistently exhibits the highest importance across all time periods (0.266–0.296), followed by White/Caucasian and Black/African American race categories. Notable temporal attenuation of demographic feature importance is observed as prediction windows narrow toward fall events, indicating the model’s adaptive reliance on proximal clinical indicators for imminent risk assessment. Female sex shows increasing importance with extended prediction horizons, while remaining demographic categories demonstrate minimal predictive contribution (SHAP < 0.01) across all timeframes

Analysis of demographic predictors across seven prediction horizons (Fig. 5) demonstrates a consistent importance hierarchy. Non-Hispanic ethnicity exhibited the highest mean SHAP values across all time periods (range = 0.266–0.296), establishing it as the most influential demographic predictor regardless of temporal distance from fall events. White/Caucasian and Black/African American race categories formed a secondary importance tier, maintaining substantial predictive contributions with mean SHAP values of 0.094–0.145 and 0.078–0.112, respectively. Female sex demonstrated increasing importance with extended prediction windows (0.048 to 0.082), contrasting with consistently negligible male sex contributions across all horizons. American Indian/Alaska Native, Asian, and Multiracial race categories, along with male sex, exhibited uniformly low predictive importance (mean SHAP < 0.01), indicating minimal contribution to model performance across all temporal frameworks.

Critically, demographic feature importance systematically declined as prediction windows approached fall events. This temporal attenuation suggests that while demographic characteristics provide foundational risk stratification for long-term assessment, proximal clinical and behavioral indicators assume greater predictive dominance in immediate pre-fall periods. The observed pattern indicates appropriate model behavior, whereby static demographic features yield predictive precedence to dynamic, time-sensitive variables as temporal proximity to adverse events increases.

To analyze the consistency of feature importance across different time horizons, we constructed a feature ranking matrix. For each of the seven prediction models, we first identified the top 40 most influential features by calculating the mean absolute SHAP value for each predictor. These features were then ranked from 1 (most important) to 40 (least important) for each specific time horizon. This process yielded a comprehensive matrix where each row represents a unique clinical feature, and each column represents a prediction window. The cells of the matrix contain the feature’s importance rank for that specific model, with a value of “N/A” indicating that the feature was not among the top 40 predictors for that timeframe. This visualization allows for a direct comparison of how the relative importance of key predictors shifts as the prediction horizon extends from the short-term to the long-term.

The feature importance rankings, presented in Table 3, reveal a dynamic clinical risk landscape where the factors predicting falls evolve significantly as the prediction horizon extends. We identified three distinct categories of predictors based on their temporal behavior: consistent, short-term specific, and long-term specific.

**Table 3:** Comparative rank of fall predictors across seven prediction windows

Disease names	3 months	6 months	12 months	24 months	36 months	48 months	60 months
Hypertension with complications and secondary hypertension	2	2	3	4	5	5	12
Other aftercare encounter	7	8	7	9	8	8	11
Nonspecific chest pain	9	10	8	8	11	10	8
Low back pain	5	5	5	6	15	16	18
Nervous system pain and pain syndromes	17	14	10	7	7	12	9
Osteoarthritis	24	25	1	11	9	11	7
Acute bronchitis	18	16	14	13	14	15	16
Superficial injury; contusion, initial encounter	8	9	9	14	17	23	27
Abnormal findings without diagnosis		27	17	15	12	9	14
Other general signs and symptoms	19	31	25	17	10	6	5
Musculoskeletal pain, not low back pain	12	11	13	12	31	25	10
Depressive disorders	10	12	12	16	19	28	26
Benign neoplasms				34	18	13	13
Osteoporosis	21	18	16	21	20	20	21
Other nervous system disorders (not hereditary or degenerative)	23	35	23	18	13	14	15
Urinary tract infections	13	13	18	20	27	33	
Neurocognitive disorders	15	19	29				
Respiratory signs and symptoms	6	7	21	37	33	21	24
Sprains and strains, initial encounter	20	17	19	24	22	29	29
Nervous system signs and symptoms	16	26	28				
Anxiety and fear-related disorders	26	22	22	26	24	24	22
Skin and subcutaneous tissue infections	22	21	24	22	25	26	28
Other specified connective tissue disease	25	24	20	19	36	22	
Secondary malignancies	31	30	26	25	21	19	20
Cataract and other lens disorders	37	34	32	28	16	17	19
Menopausal disorders						32	23
Cardiac dysrhythmias	29	23	31	27			
Essential hypertension		20		38	29		25
Nutritional deficiencies						27	30
Noninfectious gastroenteritis	30	32	27	23	26	31	31
Urinary incontinence	28	28	34				
Disorders of lipid metabolism							32
Respiratory failure; insufficiency; arrest		40	33	30	30	30	33
Other and ill-defined cerebrovascular disease	35	33	36	31	32	34	35
Circulatory signs and symptoms				40	28		
Other specified and unspecified disorders of the ear				35	35.0		
General sensation/perception signs and symptoms	32	39					
Skin/Subcutaneous signs and symptoms							36.0
Syncope	33	36	39				
Epilepsy; convulsions	36	38	37	36	34	36	37
Abdominal pain and other digestive/abdomen signs and symptoms	34	37	38				
Nausea and vomiting					38	35	
Oculofacial plastics and orbital conditions	38		40	32	37	37	39
Contact dermatitis							38
Muscle disorders						38	
Gastritis and duodenitis				39	40	40	34
Spondylopathies/spondyloarthropathy (including infective)						39	
Alcohol-related disorders	39						
Schizophrenia spectrum and other psychotic disorders	40						
Thyroid disorders							40

**Consistent core predictors**: Several features demonstrated high and stable importance across all seven-time horizons. Demographic factors were foundational, with ethnicity non-Hispanic, race White or Caucasian, and race Black or African American consistently ranking within the top 4 predictors. This indicates a baseline risk profile tied to demographic groups that is persistent over time. Similarly, hypertension with complications and musculoskeletal pain remained potent predictors across all periods, highlighting the constant risk posed by major chronic conditions. Notably, sex female showed a clear trend of increasing importance, rising from rank 10 in the short term to rank 4 in the long term, suggesting that the risk associated with being female accumulates or becomes more pronounced over many years.

**Short-term predictors**: A distinct set of features showed high importance in the short term (3–12 months) before their predictive power waned significantly over longer horizons. These were often related to acute conditions or transient health shocks. For example, low back pain ranked 5th at 3 months but fell to 18th at 60 months. Superficial injury, contusion, and urinary tract infections were also prominent short-term predictors that faded or disappeared entirely from the top rankings in the long term. This pattern suggests these factors potently predict an imminent fall, likely due to acute pain, temporary mobility impairment, or systemic illness, but their predictive signal diminishes as the patient recovers.

**Long-term predictors**: In contrast, other features emerged as significant long-term predictors, either appearing for the first time or steadily climbing in rank over the 24- to 60-month horizons. These factors typically reflect chronic, degenerative processes or the cumulative burden of disease. Osteoarthritis, for instance, rose in importance over time, consistent with its progressive nature. More strikingly, benign neoplasms and secondary malignancies were not highly ranked initially but became top 20 predictors in the long term, likely reflecting the long-term impact of these conditions on overall health and frailty. Similarly, vague diagnostic codes like abnormal findings without diagnosis and other general signs and symptoms climbed dramatically in rank over time, suggesting that an accumulation of undiagnosed or poorly defined health issues is a strong indicator of long-term fall risk.

#### Analysis of timeframe-specific predictors

Our analysis identified distinct sets of predictors specific to either short-term or long-term fall risk, highlighting the model’s ability to differentiate between acute triggers and chronic vulnerabilities. Table 4 shows the time-specific features for short-term and long-term.

**Table 4:** Short- and long-term specific predictors

Short-term specific	Long-term specific
Abdominal pain and other digestive/abdomen signs and symptoms	Thyroid disorders
Nervous system signs and symptoms	Menopausal disorders
Syncope	Benign neoplasms
Urinary incontinence	Circulatory signs and symptoms
Schizophrenia spectrum and other psychotic disorders	Gastritis and duodenitis
Neurocognitive disorders	Nutritional deficiencies
Alcohol-related disorders	Other specified and unspecified disorders of the ear
General sensation/perception signs and symptoms	Skin/subcutaneous signs and symptoms
	Contact dermatitis
	Disorders of lipid metabolism
	Spondylopathies/spondyloarthropathy (including infective)
	Nausea and vomiting
	Muscle disorders

#### Short-term predictors: markers of acute physiological disruption

The features that predicted short-term falls are representative of acute medical events and sudden changes in a person’s condition. Factors such as syncope (fainting), abdominal pain, and various nervous system symptoms clearly point to immediate physiological distress. Furthermore, conditions that affect mental state, such as neurocognitive disorders, schizophrenia spectrum disorders, and alcohol-related disorders, were key short-term predictors. The inclusion of urinary incontinence also points to an acute issue that can temporarily and significantly increase fall risk.

#### Long-term predictors: indicators of chronic decline and cumulative deficit

In contrast, the long-term predictors were characteristic of chronic conditions and gradual decline. Conditions such as spondylopathies (spinal disorders), nutritional deficiencies, muscle disorders, and thyroid disorders represent processes that impact musculoskeletal strength and overall health over many years. Other predictors like disorders of lipid metabolism and the presence of benign neoplasms also identify a patient on a trajectory of increasing vulnerability. The inclusion of disorders of the ear further supports this, as progressively worsening balance is a classic long-term risk factor. These features do not signal an imminent fall but rather a slow accumulation of risk over time.

#### High level clinical risk factors

**Fig. 6:**
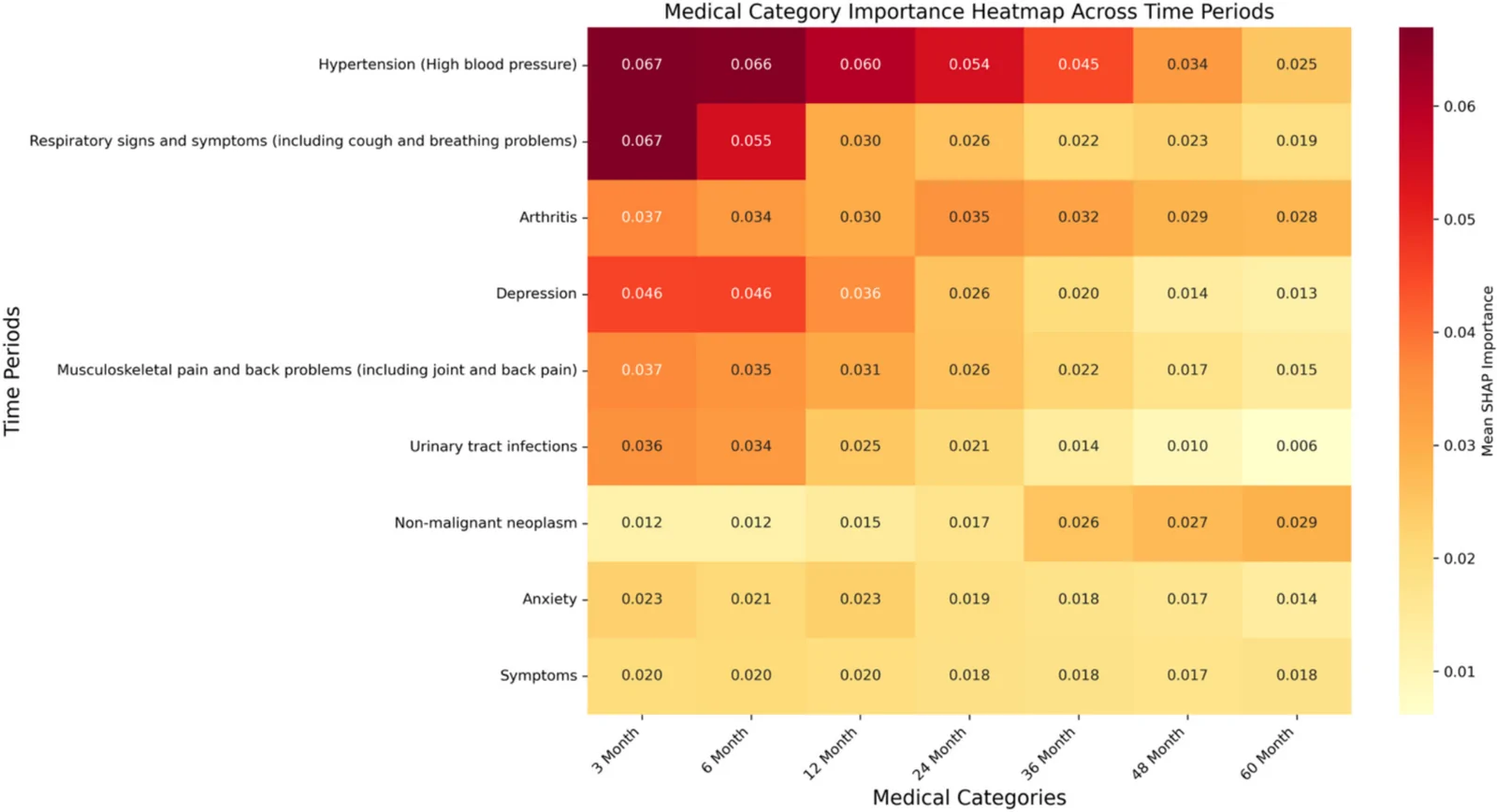
MEPS trajectory analysis: heatmap of medical category importance across prediction horizons (3–60 months) for fall risk. The color intensity reflects relative importance values, with acute conditions (e.g., respiratory, digestive, musculoskeletal pain) dominating short-term horizons, while chronic disorders (e.g., hypertension, urinary tract issues, neoplasms) show sustained or increasing influence at longer horizons

To provide a more interpretable, high-level view of clinical risk factors, we aggregated the numerous specific diagnostic codes into broader medical categories using Medical Expenditure Panel Survey (MEPS) [30]. The resulting feature importance for these categories was then analyzed across all time horizons, revealing distinct temporal patterns for chronic and acute conditions, as shown in the heatmap in Fig. 6.

This heatmap reveals that the intensity and nature of predictive signals change significantly across the prediction horizons. The models’ forecasting falls in the short term, particularly at 3 and 6 months, and is heavily influenced by categories representing acute issues. For instance, Respiratory signs and symptoms demonstrate their highest predictive importance in the short term before fading significantly over longer periods. Depression and Musculoskeletal pain follow a similar pattern, showing their greatest impact within the first year. In contrast, while these acute signals diminish, the 60-month model retains its predictive power by focusing on different factors. The importance of chronic conditions like Hypertension remains consistently high across all timeframes, while the influence of Non-malignant neoplasm steadily grows, more than doubling its importance from the 3-month to the 60-month model. This illustrates that the short-term models are tuned to high-intensity, immediate health problems, whereas the long-term model is uniquely valuable for detecting the cumulative risk of slowly developing chronic conditions.

### Survival analysis validation of temporal predictor patterns

To validate the temporal specificity of identified short-term and long-term predictors, we conducted Kaplan-Meier survival analyses examining time-to-fall relationships for key features from each category. This analysis aimed to determine whether the observed temporal patterns in model importance truly reflected distinct hazard profiles rather than statistical artifacts.

#### Short-term condition survival patterns

**Fig. 7:**
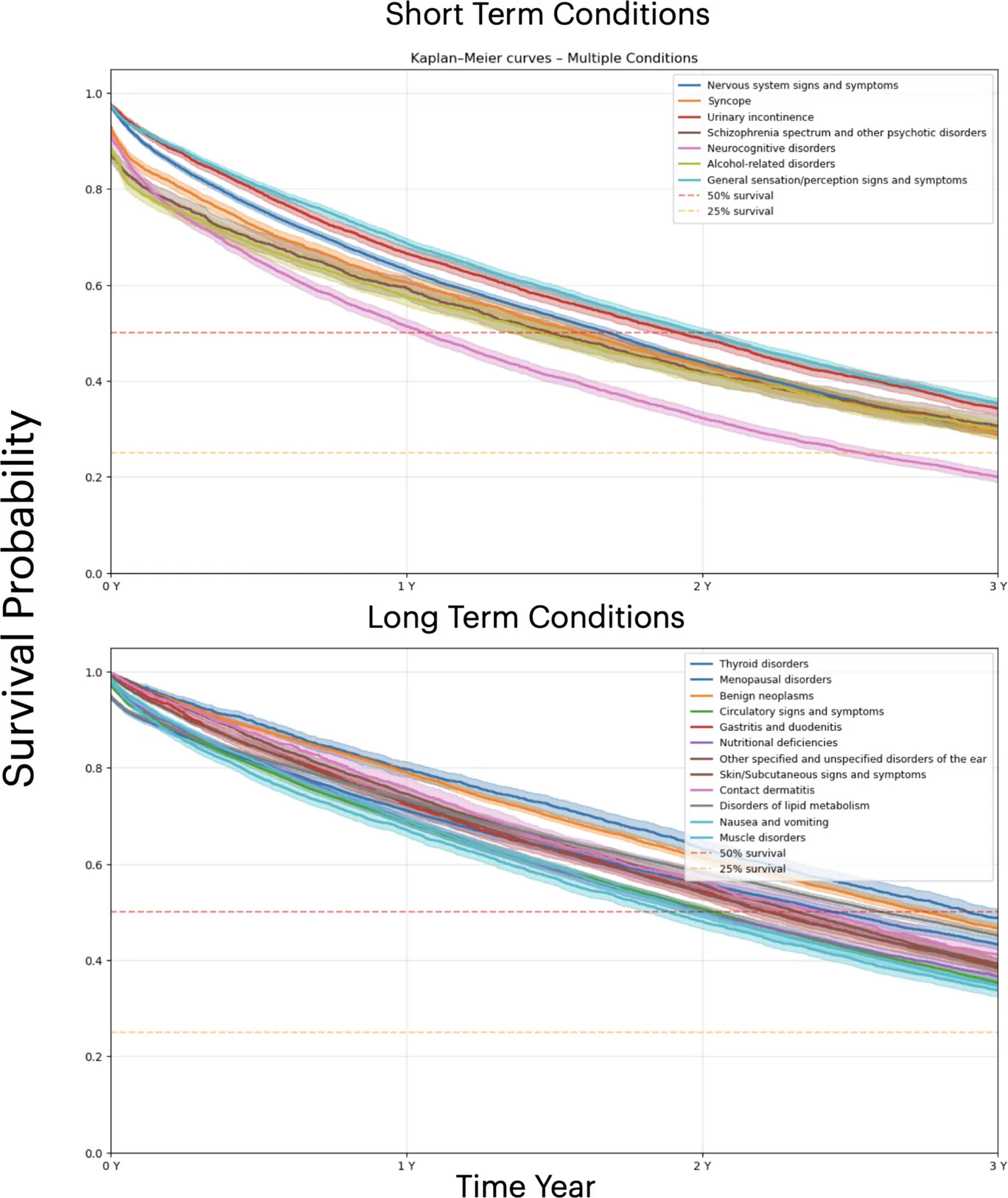
Kaplan-Meier survival analysis validates temporal specificity of fall predictors. Upper panel: Short-term specific conditions demonstrate rapid hazard acceleration with steep survival curve decline in early observation periods. Acute conditions, including schizophrenia spectrum disorders, neurocognitive disorders, and alcohol-related disorders, cross the 50% survival threshold within 12–18 months, validating their classification as immediate fall precipitants. Lower panel: Long-term specific conditions exhibit gradual, sustained decline patterns consistent with chronic progressive risk. Conditions such as thyroid disorders, nutritional deficiencies, and benign neoplasms maintain higher early survival probabilities before demonstrating steady decline over 2–3 years, confirming their role as cumulative risk factors. Dashed horizontal lines indicate 50% and 25% survival thresholds. The distinct survival trajectories provide independent validation of ML model temporal risk stratification, supporting horizon-specific fall prediction strategies

Kaplan-Meier curves for short-term specific conditions demonstrate markedly different survival trajectories (Fig. 7, upper panel). Acute conditions, including nervous system signs and symptoms, syncope, and urinary incontinence, exhibit steep initial decline curves, with survival probabilities dropping rapidly within the first year. Notably, schizophrenia spectrum and other psychotic disorders, neurocognitive disorders, and alcohol-related disorders show particularly accelerated hazard rates in early time periods, with survival curves crossing the 50% threshold within 12–18 months. This pattern validates the model’s identification of these features as short-term predictors, confirming their association with elevated immediate fall risk that diminishes over extended observation periods.

#### Long-term condition survival patterns

Conversely, long-term specific predictors exhibit more gradual, sustained decline patterns (Fig. 7, lower panel). Conditions such as thyroid disorders, menopausal disorders, and benign neoplasms demonstrate relatively preserved survival probabilities in early periods, with curves maintaining higher values through the first year before showing consistent, steady decline over 2–3 years. Nutritional deficiencies, gastritis and duodenitis, and disorders of lipid metabolism follow similar trajectories, supporting their classification as chronic, progressive risk factors rather than acute precipitants.

#### Aggregate survival analysis of temporal predictor categories

To evaluate the overall temporal risk patterns between condition categories, we performed a comparative survival analysis aggregating all short-term and long-term specific predictors into composite groups. This merged analysis provides a comprehensive view of the fundamental differences in hazard profiles between acute and chronic fall risk factors.

**Fig. 8:**
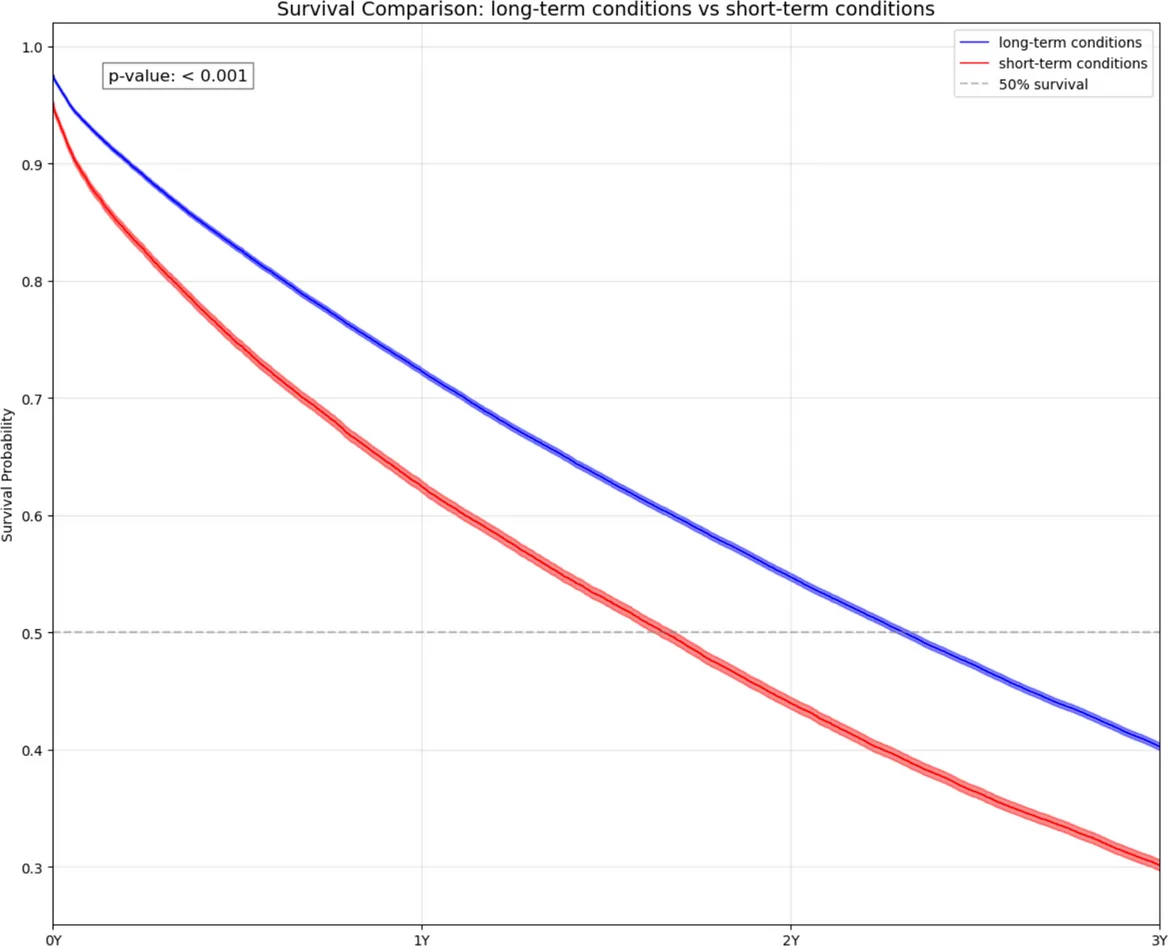
Aggregate survival analysis demonstrates distinct temporal risk profiles between predictor categories. Kaplan-Meier curves comparing composite groups of short-term specific conditions (red) versus long-term specific conditions (blue) over 3-year follow-up period. Short-term conditions exhibit consistently higher hazard rates with 50% survival at approximately 18 months, compared to 24 months for long-term conditions. The sustained separation between curves (p < 0.001, log-rank test) validates the fundamental distinction between acute precipitating factors and chronic predisposing conditions for fall risk. Dashed horizontal line indicates 50% survival threshold. The significant survival differential supports implementation of temporally tailored clinical intervention strategies based on condition category

The aggregate Kaplan-Meier analysis reveals statistically significant differences between short-term and long-term predictor groups (*p* < 0.001, log-rank test; Fig. 8). Short-term conditions demonstrate consistently higher hazard rates across all time periods, with survival probability declining rapidly from onset and maintaining lower survival throughout the 3-year observation period. The short-term condition curve crosses the 50% survival threshold at approximately 18 months, compared to 26 months for long-term conditions.

Notably, while both groups show declining survival over time, the hazard differential remains consistent rather than converging, indicating that short-term predictors represent fundamentally higher-risk phenotypes rather than simply earlier manifestations of the same underlying risk processes. The sustained separation between curves validates the clinical distinction between acute precipitating factors and chronic predisposing conditions in fall risk assessment.

This aggregate analysis confirms that patients presenting with short-term specific conditions require immediate, intensive fall prevention interventions given their elevated baseline hazard. Conversely, patients with long-term specific conditions benefit from sustained, longitudinal risk management strategies. The significant survival differential (*p* < 0.001) provides robust statistical support for implementing temporally tailored clinical protocols based on the predictive model’s risk stratification framework.

## Discussion

This study represents a significant advancement in fall risk prediction research by moving beyond the static, single-timeframe analysis of previous ML approaches to instead model the dynamic evolution of fall risk over longer periods. Our multi-horizon approach reveals fundamental insights into the temporal dynamics of fall risk that have profound implications for personalized prevention strategies. We identified three primary temporal patterns: (1) demographic features (particularly non-Hispanic ethnicity) maintain consistent importance across all horizons but decrease as fall events approach, indicating the model’s appropriate shift toward proximal clinical indicators; (2) short-term predictors including respiratory symptoms, depression, and musculoskeletal pain demonstrate peak importance within 3–12 months, reflecting acute precipitating conditions; and (3) long-term predictors such as non-malignant neoplasms, hypertension, and progressive musculoskeletal disorders show increasing importance over extended horizons, representing cumulative chronic risk factors. Survival analysis validated these temporal distinctions, with short-term conditions exhibiting earlier time-to-fall compared to long-term conditions (*p* < 0.001).

### Interpretation of results

#### Temporal evolution of predictive performance

Our findings demonstrate a systematic trade-off in model performance across prediction horizons that reflects the underlying nature of fall risk. Short-term models (3–12 months) achieved balanced discrimination with AUCs of 0.70–0.72, comparable to the best-performing models in recent literature. However, the progressive increase in sensitivity from 67% at 3 months to 80% at 60 months, coupled with declining specificity (65 to 46%), reveals that longer-term models become increasingly effective at identifying individuals who will eventually fall, albeit with higher false-positive rates.

This performance pattern challenges the conventional assumption that longer prediction windows inherently reduce accuracy. Instead, our results suggest that different time horizons capture fundamentally different aspects of fall risk. Short-term models excel at identifying imminent threats with balanced performance, while long-term models serve a complementary role by casting a wider net to capture individuals on trajectories toward increased vulnerability.

#### Clinical alignment of temporal risk patterns

The identified temporal patterns demonstrate remarkable alignment with established clinical knowledge of fall pathophysiology, validated by the specific ranking evolution across prediction horizons. The most striking finding of our analysis is the clear differentiation between acute triggers and chronic vulnerabilities revealed through SHAP analysis across time horizons. This temporal stratification of risk factors represents a paradigm shift from static risk profiling to dynamic risk understanding.

#### Short-term specific predictors: acute precipitating events

Several conditions appear exclusively or predominantly in short-term prediction models (3–12 months), representing acute medical events that directly precipitate falls through immediate physiological disruption. Syncope exemplifies this pattern, appearing only in short-term models, representing acute cerebrovascular or cardiovascular events causing immediate consciousness impairment and fall risk [31–33]. Nervous system signs and symptoms demonstrate similar behavior, appearing exclusively at short horizons, capturing acute neurological deterioration, delirium, or medication effects that impair balance and cognition [34–37].

Neurocognitive disorders show classic short-term specificity (15th–29th at 3–12 months only), likely representing acute confusional states and delirium rather than chronic dementia, aligning with clinical observations that delirium is a major precipitant of hospital falls [36, 37]. Urinary incontinence appears predominantly in short-term models (28th–34th), likely reflecting acute conditions such as urinary tract infections that cause both urinary urgency and potential delirium in elderly patients [36, 37]. Moreover, urinary incontinence accompanied by delirium or mental status changes can also be indicative of Normal Pressure Hydrocephalus (NPH), a condition characterized by the clinical triad of gait abnormalities, cognitive impairment, and urinary incontinence, which substantially elevates fall risk [38].

Abdominal pain and digestive symptoms (34th–38th in short-term models) indicate acute gastrointestinal conditions causing pain, medication effects, or electrolyte disturbances that immediately impair mobility, with conditions like syncope from GI bleeding or dehydration and musculoskeletal pain from referred abdominal pathology further contributing to fall risk [27, 39, 40]. In addition, some abdominal pain medications such as proton pump inhibitors (PPIs) can be associated with fall as well [41]. Alcohol-related disorders (39th at 3 months only) and schizophrenia spectrum disorders (40th at 3 months only) represent acute intoxication or psychiatric decompensation states that severely compromise judgment and coordination [42–45]. General sensation/perception signs and symptoms (32nd–39th in short-term models) capture acute sensory disturbances such as dizziness that immediately affect balance and spatial orientation [46, 47].

#### Long-term specific predictors: chronic cumulative risk factors

Conversely, long-term specific conditions appear exclusively or achieve significance only in extended prediction windows beyond 12 months, reflecting chronic processes that accumulate fall risk through gradual physiological decline. Thyroid disorders, representing chronic endocrine dysfunction, cause gradual muscle weakness, bone density loss, and cardiovascular effects that increase fall risk over the years [48–50].

Nutritional deficiencies (27th–30th in long-term models only) contribute to osteoporosis, muscle weakness, and peripheral neuropathy through chronic vitamin D and B12 deficiency processes [51–55]. Spondylopathies (39th at 60 months only) represent progressive spinal disorders that gradually impair mobility, balance, and cause chronic pain, with fall risk accumulating as structural changes advance [56–58].

Menopausal disorders appear in mid-to-long-term models (23rd–32nd beyond 12 months), capturing the progressive effects of estrogen deficiency on bone density, muscle mass, and balance over years [59–62]. Benign neoplasms, notably vestibular schwannoma, spinal meningioma, and pituitary adenoma, can elevate fall risk through vestibular dysfunction, hydrocephalus, myelopathic gait disturbance, and tumor-related visual field loss, respectively, consistent with clinically observable balance and mobility impairment [63–68].

Gastritis and duodenitis are GI illnesses that may involve bleeding and vomiting, potentially leading to anemia, orthostatic hypotension, electrolyte imbalance, and dehydration, as well as medication-related effects (e.g., PPIs), all of which immediately increase fall risk [41, 69–72]. Disorders of lipid metabolism represent chronic metabolic dysfunction, linked to cerebrovascular disease, frailty, and muscle weakness, and may also interact with statin-associated myopathy, thereby contributing to fall risk over longer horizons [73–75].

#### Clinical reality and implementation implications

The alignment between our temporal risk patterns and clinical pathophysiology supports the development of horizon-specific intervention strategies. Short-term high-risk patients identified through acute predictors (e.g., respiratory symptoms, depression, acute pain) require immediate, intensive interventions including medication reconciliation, acute symptom management, and enhanced environmental safety measures. Conversely, patients identified through long-term predictors benefit from longitudinal management strategies addressing chronic disease progression, functional preservation, and proactive risk factor modification.

This temporal risk stratification framework addresses a critical gap in current clinical practice, where fall risk assessment often employs static screening tools that fail to account for the dynamic nature of risk factors. By distinguishing between immediate precipitating factors and gradual predisposing conditions, clinicians can align intervention intensity and duration with the underlying temporal risk profile, potentially improving both prevention effectiveness and resource allocation efficiency.

#### Comparison with existing literature

Most existing fall prediction models employ single time horizons, typically focusing on near fall prediction windows [13, 16, 17, 19–21, 26]. While these studies have identified important risk factors, including cognitive impairment, polypharmacy, and mobility limitations, they lack the temporal granularity to distinguish between acute precipitating factors and chronic predisposing conditions.

The few studies that have examined multiple prediction horizons have primarily focused on model performance comparison rather than extracting clinical insights from temporal patterns. One example is a longitudinal cohort study of 5492 residents across 27 aged care facilities in Sydney, Australia, which applied a landmarking approach with monthly prediction windows over 60 months. The authors developed dynamic fall risk models separately for residents with and without dementia, identifying 15 predictors in the dementia cohort and 12 in the nondementia cohort, with prior falls emerging as the strongest predictor in both groups (hazard ratio (HR) ~4–5). Model performance remained moderate to strong across horizons, with AUC values typically between 0.75 and 0.85, and the team translated regression coefficients into point-based scoring systems. While the study demonstrated the feasibility of dynamic prediction across multiple horizons, its primary focus was on internal validation and risk score development, rather than interpretation of how predictors may vary over time [11].

Our approach advances beyond existing literature by systematically analyzing feature importance evolution across multiple horizons and validating temporal patterns through survival analysis. This methodology reveals that effective fall prediction requires temporally adaptive risk assessment, where short-term models prioritize acute clinical indicators while long-term models emphasize cumulative chronic conditions.

### Temporal patterns in predicting fall

**Fig. 9:**
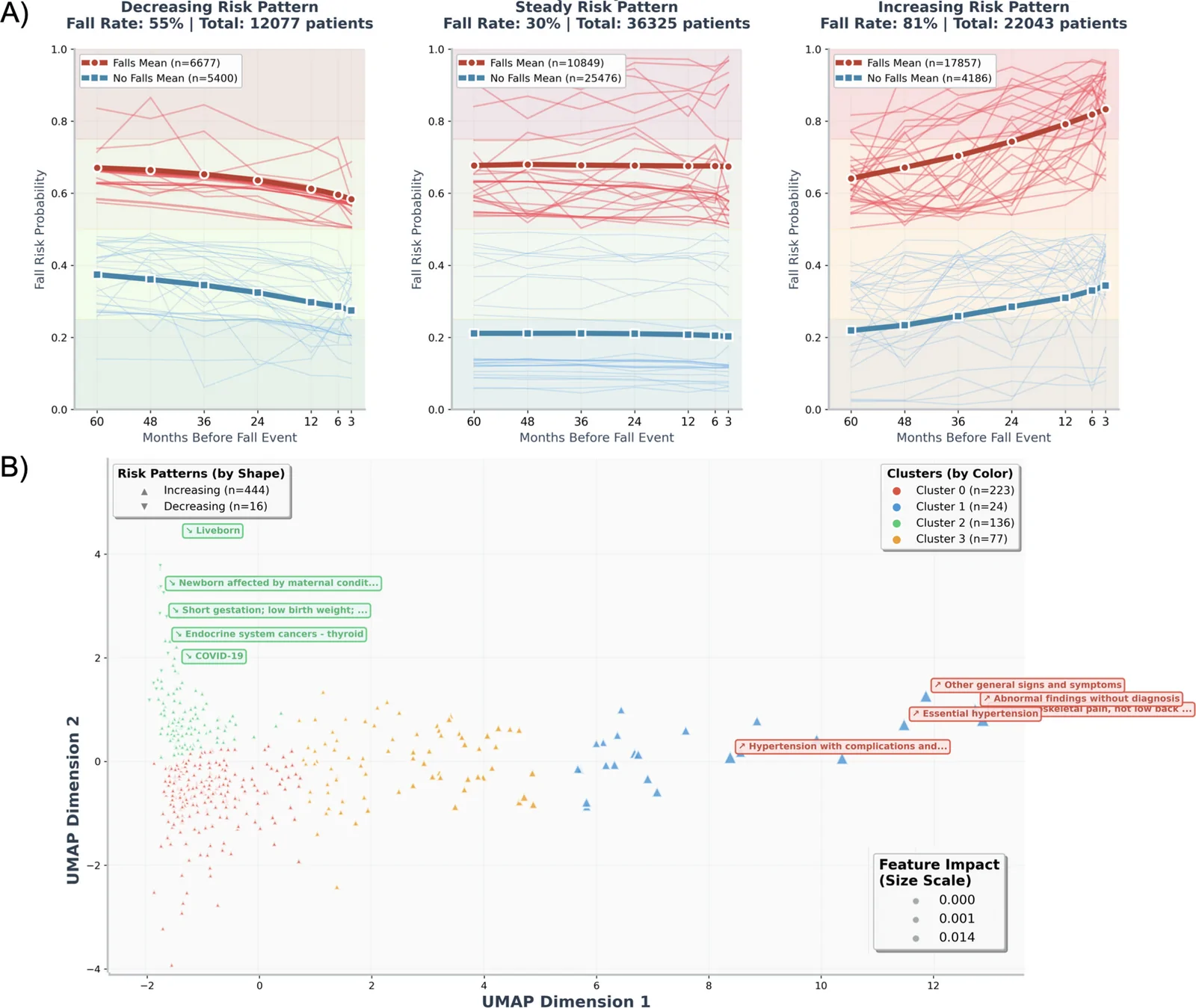
Risk trajectory patterns reveal distinct temporal phenotypes in fall prediction. **A** Individual patient risk trajectories classified into three patterns based on slope analysis across 60–3 months before fall events, analyzing 77,445 correctly predicted patients. Decreasing risk pattern (left, *n* = 12,077, 55% fall rate) shows declining probabilities from 0.68 to 0.57 in fall patients (red thick line) and 0.38 to 0.28 in non-fall patients (blue thick line). Steady risk pattern (center, *n* = 36,325, 30% fall rate) maintains consistent probabilities around 0.66–0.68 for fall patients and 0.20–0.21 for non-fall patients. Increasing risk pattern (right, *n* = 22,043, 81% fall rate) demonstrates escalating probabilities from 0.65 to 0.83 in fall patients and 0.23 to 0.33 in non-fall patients. Thin lines represent individual patient trajectories (sample of 30 per group), thick lines show group means with 95% confidence intervals. **B** UMAP dimensionality reduction of trajectory-defining features reveals biological clustering corresponding to risk patterns. Decreasing risk patients (downward triangles) cluster around acute recoverable conditions (COVID-19, maternal conditions), while increasing risk patients (upward triangles) associate with progressive chronic conditions (essential hypertension, skeletal pain, abnormal findings). Point size reflects feature impact magnitude. Four distinct clusters emerge, validating the biological basis of temporal risk evolution patterns. This trajectory-based stratification provides prognostic information beyond static risk assessment, enabling temporally adaptive intervention strategies

To understand the dynamic evolution of fall risk in individual patients, we conducted a comprehensive trajectory analysis examining how predicted probabilities changed across time horizons for correctly predicted patients. This analysis focused exclusively on patients whose fall outcomes were accurately predicted across all seven-time periods, ensuring reliable trajectory characterization (Fig. 9A). Through systematic tracking of individual patient risk scores across all prediction horizons, we identified three primary groups: patients with decreasing risk patterns, patients with steady risk patterns, and patients with increasing risk patterns. The decreasing risk pattern showed fall probabilities declining from approximately 0.7 at 60 months to ~0.55 at 3 months before the event, while the increasing risk pattern demonstrated the opposite trajectory, with probabilities rising from 0.6 to 0.8 as the fall event approached. The steady risk pattern maintained relatively constant probabilities around 0.6–0.7 across all time horizons, suggesting a persistent baseline vulnerability.

We applied Uniform Manifold Approximation and Projection (UMAP) dimensionality reduction to visualize how different feature patterns clustered according to risk trajectory groups (Fig. 9B). The UMAP analysis revealed distinct feature clusters that corresponded to these risk patterns, with clear separation between patients following different trajectory paths. Features contributing to decreasing risk trajectories clustered around markers of recovering health status and resolved acute conditions, while those driving increasing risk patterns were associated with progressive chronic conditions, accumulating comorbidities, and markers of declining functional status. This visualization demonstrates that the temporal evolution of fall risk reflects meaningful underlying changes in patient health status, providing biological plausibility for our multi-horizon modeling approach and suggesting that different trajectory patterns may require fundamentally different intervention strategies.

The trajectory analysis demonstrates markedly different fall risk profiles between pattern groups. The increasing risk pattern shows the highest fall rate (81%), indicating that escalating risk trajectories are highly predictive of adverse outcomes. This contrasts with steady risk patterns (30% fall rate) and decreasing risk patterns (55% fall rate), suggesting that the temporal direction of risk change provides substantial prognostic information beyond static risk assessment.

The feature clustering reveals that decreasing risk patterns associate with resolution of acute conditions (COVID-19 recovery, post-surgical states, resolved pregnancy complications), while increasing patterns link to progressive chronic disease accumulation. This biological plausibility supports the clinical validity of trajectory-based risk stratification.

These trajectory-specific patterns are consistent with prior clinical and epidemiologic evidence on fall mechanisms. The decreasing risk group, enriched for conditions such as COVID-19 recovery, post-surgical states, and pregnancy-related complications, mirrors studies showing that falls often cluster in high-risk periods and then decline as acute stressors resolve. For example, perioperative cohorts report that most post-surgical falls occur in the early weeks to months after surgery, with a gradual reduction as patients recover strength and mobility [28]. Similarly, work on COVID-19 has documented both balance impairment and increased fall risk after infection, but also short-term changes in fall incidence when mobility is abruptly restricted, as seen in the McIntyre, Cecily C et al. [76] study where stay-at-home restrictions were associated with fewer falls among high-risk older adults [76, 77]. Pregnancy-related pelvic girdle pain and pregnancy-induced gait changes have likewise been linked to transient increases in fall risk that typically improve postpartum, aligning with our observation of resolving risk in this subgroup [78, 79]. In contrast, our increasing risk trajectories, characterized by progressive accumulation of chronic diagnoses, are consistent with literature showing that multimorbidity patterns and frailty trajectories are strongly associated with recurrent and injurious falls over time [80–82]. Together, these convergences between our trajectory groups and known clinical patterns support the biological plausibility of the temporal profiles identified by our multi-horizon models.

This trajectory-based risk stratification framework enables personalized intervention timing and intensity. Patients with increasing risk patterns require escalating preventive measures as fall events approach, while those with decreasing patterns may benefit from maintenance strategies to preserve recovery gains. Steady risk patients need consistent long-term management approaches. The ability to predict not just fall risk but its temporal evolution represents a significant advance in precision fall prevention.

### Clinical implications and future directions

#### Transforming prevention strategies

Our findings suggest a paradigm shift toward temporally informed fall prevention. Short-term high-risk individuals identified by acute predictors require immediate interventions: urgent medication reviews, home safety assessments, and intensive monitoring. Conversely, long-term high-risk individuals benefit from sustained interventions addressing chronic conditions: physical therapy, nutritional counseling, and management of progressive diseases. This temporal approach could dramatically improve the efficiency of fall prevention programs by matching intervention intensity and duration to risk timeframe. Rather than applying uniform interventions to all “high-risk” patients, clinicians can now tailor approaches based on whether risk stems from acute vulnerabilities or chronic decline.

#### Foundation for personalized medicine

Our multi-horizon modeling framework provides a foundation for truly personalized fall prevention. By understanding how individual risk factors evolve over time, clinicians can develop patient-specific monitoring schedules and intervention plans. For instance, a patient with prominent short-term predictors might require frequent reassessment over 6–12 months, while those with long-term risk factors need sustained management over several years. The predictive value of seemingly minor conditions in long-term models also highlights the importance of comprehensive geriatric assessment and the potential role of artificial intelligence in synthesizing complex, multidimensional health information that might otherwise be overlooked.

### Study limitations

Several limitations should be acknowledged. First, our retrospective design, while enabling comprehensive temporal analysis, cannot establish causality between identified predictors and fall outcomes. The reliance on EHR data may introduce documentation bias and miss important clinical nuances not captured in structured data fields. In particular, our outcome definition is based on ICD-10 fall codes recorded within a single health system, which likely underascertains non-injurious or out-of-system falls. As a result, the model is effectively calibrated to predict medically attended falls, that is, fall events severe enough to generate clinical encounters and billing codes. Although this may miss minor, unreported falls, medically attended falls constitute the subset of events with the greatest clinical and economic burden and are therefore a high-priority target for health system intervention.

Second, despite 1:1 matching on age and sex, the observed associations between race/ethnicity and fall risk raise the possibility of residual confounding and health system–specific bias. It is likely that part of the predictive signal attributed to ethnicity reflects underlying socioeconomic conditions, neighborhood-level factors, and patterns of healthcare utilization within the catchment area, rather than intrinsic biological susceptibility. Moreover, our data are drawn from a single academic health system, which may limit generalizability to other regions, care models, and demographic compositions. Future work should include external validation across diverse populations and settings, along with systematic subgroup performance assessment, to disentangle biological risk from structural and access-related factors and to evaluate the fairness of any potential clinical deployment.

### Research contributions and future impact

Future research should focus on prospective validation of our temporal risk stratification approach, development of intervention protocols aligned with different risk timeframes, and extension of multi-horizon modeling to other age-related health outcomes. A key direction for refinement is the integration of multimodal data streams with our current EHR-based framework. Incorporating richer medication information (e.g., polypharmacy burden, high-risk drug classes, and adherence patterns) and unstructured clinical notes could enhance the capture of evolving clinical status that is not fully reflected in diagnostic codes alone. In parallel, combining these longitudinal EHR features with real-time monitoring data, such as wearable sensor–derived measures of gait variability and activity patterns, would allow future models to jointly represent systemic physiological vulnerability and immediate mechanical instability. Such multimodal, dynamically updated models could support continuous risk monitoring, enable timely adjustment of prevention strategies, and provide a more holistic view of fall risk across the adult life course.

## Conclusion

This study addresses a critical gap in fall risk prediction by demonstrating that fall risk is a dynamic phenomenon requiring temporal-specific approaches rather than a single, static assessment. Through comprehensive analysis across seven prediction horizons (3–60 months), we revealed distinct categories of risk factors: acute triggers dominating short-term predictions (syncope, acute pain) versus chronic vulnerabilities emerging in long-term models (nutritional deficiencies, spondylopathies, progressive conditions). Our SHAP analysis showed that while short-term models achieve balanced discrimination (AUC ~0.70–0.72) and excel at identifying imminent threats, long-term models progressively favor sensitivity (reaching 80% at 60 months) to capture individuals on trajectories toward increased vulnerability. This temporal differentiation provides the first data-driven framework for understanding when and why patients are at risk, moving beyond binary risk classifications to nuanced risk profiling that directly informs intervention timing and intensity.

The clinical implications of these findings are transformative for fall prevention practice. Rather than applying uniform interventions to all “high-risk” patients, our temporal framework enables personalized prevention strategies where patients with acute triggers receive immediate interventions (urgent medication reviews, home safety assessments) while those with chronic vulnerabilities benefit from sustained management (physical therapy, nutritional counseling). It is important to acknowledge that while our study population spanned a broad age range (median 57 years) rather than a strictly geriatric cohort, this distribution supports a life-course approach to prevention. By capturing risk factors in middle-aged adults, our findings offer a pathway to identify and mitigate early disease trajectories before they culminate in the complex frailty typically seen in older populations. This approach represents a paradigm shift from reactive, episodic care to proactive, temporally informed prevention that aligns resource allocation with risk timeframes. Beyond fall prediction, our multi-horizon modeling framework establishes a methodological foundation for temporal health prediction applicable to other geriatric syndromes, ultimately advancing toward precision medicine in geriatric care where AI-assisted systems enhance clinical judgment to improve outcomes for millions of older adults at risk worldwide.

## Supplementary information

Below is the link to the electronic supplementary material.

ESM 1 (DOCX 4.40 MB)

## Data Availability

The data that support the findings of this study are not publicly available due to institutional restrictions on electronic health record data but may be available from the corresponding author on reasonable request and subject to institutional approval.
