# Supplementary material for: Explainable AI reveals temporal risk pathways in fall prediction: Extracting clinical insights from multi-horizon machine learning models
Source: GeroScience. 2026 Feb 4;48(6):8299–324. doi: 10.1007/s11357-026-02117-x (PMC13638931; doi:10.1007/s11357-026-02117-x)
Supplement: Supplementary file 1 [file 11357_2026_2117_MOESM1_ESM.docx]

**Appendix 1**

**3 Month Results**

Table 1: 3-month models

| **Model name** | **Accuracy** | **Precision** | **F1-Score** | **AUC** | **Sensitivity** | **Specificity** |
| --- | --- | --- | --- | --- | --- | --- |
| logistic regression | 65.00 | 0.6560 | 0.6401 | 0.7250 | 0.6250 | 0.6748 |
| decision tree | 62.05 | 0.6095 | 0.6349 | 0.6183 | 0.6626 | 0.5787 |
| random forest | **68.27** | 0.6578 | **0.7036** | 0.7473 | **0.7562** | 0.6097 |
| gradient boosting | 65.75 | 0.6747 | 0.6369 | 0.7256 | 0.6030 | 0.7115 |
| Ada boost | 64.46 | 0.6437 | 0.6426 | 0.7126 | 0.6415 | 0.6477 |
| extra trees | 67.54 | 0.6508 | 0.6976 | 0.7348 | 0.7515 | 0.5999 |
| XGBoost | 67.87 | **0.6921** | 0.6645 | **0.7581** | 0.6391 | **0.7180** |

Figure 1: 3-Month AUC

**6 Month Results**

Table 2: 6 month table

| **Model name** | **Accuracy** | **Precision** | **F1-Score** | **AUC** | **Sensitivity** | **Specificity** |
| --- | --- | --- | --- | --- | --- | --- |
| logistic regression | 64.52 | 0.6477 | 0.6390 | 0.7183 | 0.6306 | 0.6596 |
| decision tree | 62.34 | 0.6101 | 0.6411 | 0.6214 | 0.6755 | 0.5716 |
| random forest | **67.76** | 0.6511 | **0.7013** | 0.7421 | **0.7600** | 0.5959 |
| gradient boosting | 64.92 | 0.6649 | 0.6285 | 0.7196 | 0.5960 | 0.7020 |
| Ada boost | 63.83 | 0.6482 | 0.6226 | 0.7055 | 0.5989 | 0.6775 |
| extra trees | 67.28 | 0.6468 | 0.6970 | 0.7293 | 0.7556 | 0.5907 |
| XGBoost | 67.54 | **0.6840** | 0.6652 | **0.7543** | 0.6474 | **0.7032** |

Figure 2: 6 Month AUC

**12 Month Results**

Table 3: 12 month

| **Model name** | **Accuracy** | **Precision** | **F1-Score** | **AUC** | **Sensitivity** | **Specificit** |
| --- | --- | --- | --- | --- | --- | --- |
| logistic regression | 63.5017 | 0.6319 | 0.6359 | 0.7066 | 0.6401 | 0.6300 |
| decision tree | 62.0307 | 0.6051 | 0.6421 | 0.6156 | 0.6839 | 0.5572 |
| random forest | **67.2916** | 0.6448 | **0.6995** | 0.7344 | **0.7642** | 0.5823 |
| gradient boosting | 63.6884 | 0.6480 | 0.6193 | 0.7084 | 0.5930 | **0.6804** |
| Ada boost | 61.6598 | 0.6196 | 0.6077 | 0.6921 | 0.5963 | 0.6367 |
| extra trees | 66.8450 | 0.6404 | 0.6961 | 0.7222 | 0.7624 | 0.5752 |
| XGBoost | 66.8248 | **0.6687** | 0.6652 | **0.7455** | 0.6616 | 0.6748 |

Figure 3

**24 Month Results**

Table 4: 24 month

| **Model name** | **Accuracy** | **Precision** | **F1-Score** | **AUC** | **Sensitivity** | **Specificity** |
| --- | --- | --- | --- | --- | --- | --- |
| logistic regression | 62.14 | 0.6094 | 0.6374 | 0.6922 | 0.6682 | 0.5750 |
| decision tree | 61.51 | 0.5982 | 0.6416 | 0.6092 | 0.6919 | 0.5389 |
| random forest | 65.99 | 0.6325 | **0.6892** | 0.7163 | **0.7572** | 0.5635 |
| gradient boosting | 63.07 | 0.6111 | 0.6572 | 0.6992 | 0.7109 | 0.5511 |
| Ada boost | 60.97 | 0.5958 | 0.6319 | 0.6768 | 0.6728 | 0.5470 |
| extra trees | 65.58 | 0.6286 | 0.6861 | 0.7045 | 0.7552 | 0.5572 |
| XGBoost | **66.26** | **0.6434** | 0.6811 | **0.7322** | 0.7234 | **0.6023** |

Figure 4

**36 Month Results**

Table 5: 36 month

| **Model name** | **Accuracy** | **Precision** | **F1-Score** | **AUC** | **Sensitivity** | **Specificity** |
| --- | --- | --- | --- | --- | --- | --- |
| logistic regression | 61.67 | 0.5955 | 0.6511 | 0.6803 | 0.7180 | 0.5162 |
| decision tree | 61.58 | 0.5970 | 0.6457 | 0.6102 | 0.7032 | 0.5291 |
| random forest | **65.49** | **0.6267** | **0.6868** | 0.7056 | 0.7596 | 0.5511 |
| gradient boosting | 62.23 | 0.5901 | 0.6761 | 0.6888 | **0.7915** | 0.4544 |
| Ada boost | 61.00 | 0.5831 | 0.6603 | 0.6646 | 0.7610 | 0.4602 |
| extra trees | 64.87 | 0.6207 | 0.6823 | 0.6961 | 0.7576 | 0.5406 |
| XGBoost | 65.20 | 0.6258 | 0.6821 | **0.7200** | 0.7495 | **0.5553** |

Figure 5

**48 Month Results**

Table 6: 48 month

| **Model name** | **Accuracy** | **Precision** | **F1-Score** | **AUC** | **Sensitivity** | **Specificity** |
| --- | --- | --- | --- | --- | --- | --- |
| logistic regression | 62.17 | 0.5909 | 0.6729 | 0.6727 | 0.7814 | 0.4633 |
| decision tree | 61.49 | 0.5928 | 0.6518 | 0.6068 | 0.7237 | 0.5068 |
| random forest | **65.00** | **0.6199** | 0.6863 | 0.7007 | 0.7686 | **0.5323** |
| gradient boosting | 61.75 | 0.5829 | 0.6797 | 0.6838 | **0.8149** | 0.4216 |
| Ada boost | 60.64 | 0.5807 | 0.6562 | 0.6584 | 0.7542 | 0.4597 |
| extra trees | 64.50 | 0.6154 | 0.6823 | 0.6885 | 0.7657 | 0.5252 |
| XGBoost | 64.98 | 0.6178 | **0.6889** | **0.7125** | 0.7787 | 0.5220 |

Figure 6

**60 Month Results**

Table 7: 60 month

| **Model name** | **Accuracy** | **Precision** | **F1-Score** | **AUC** | **Sensitivity** | **Specificity** |
| --- | --- | --- | --- | --- | --- | --- |
| logistic regression | 62.12 | 0.5852 | 0.6839 | 0.6668 | 0.8226 | 0.4214 |
| decision tree | 61.55 | 0.5921 | 0.6550 | 0.6062 | 0.7327 | 0.4993 |
| random forest | **64.82** | **0.6173** | 0.6861 | 0.6923 | 0.7722 | **0.5251** |
| gradient boosting | 61.91 | 0.5824 | 0.6848 | 0.6799 | 0.8309 | 0.4090 |
| Ada boost | 60.92 | 0.5696 | **0.6917** | 0.6531 | **0.8803** | 0.3402 |
| extra trees | 64.18 | 0.6116 | 0.6813 | 0.6820 | 0.7690 | 0.5155 |
| XGBoost | 64.53 | 0.6120 | 0.6882 | **0.7055** | 0.7860 | 0.5056 |

Figure 7
